# Vaccines Against Antimicrobial Resistance

**DOI:** 10.3389/fimmu.2020.01048

**Published:** 2020-06-03

**Authors:** Roberto Rosini, Sonia Nicchi, Mariagrazia Pizza, Rino Rappuoli

**Affiliations:** ^1^GSK, Siena, Italy; ^2^Department of Pharmacy and Biotechnology (FaBiT), University of Bologna, Bologna, Italy; ^3^vAMRes Lab, Toscana Life Sciences, Siena, Italy; ^4^Faculty of Medicine, Imperial College London, London, United Kingdom

**Keywords:** vaccines, infectious diseases, antimicrobial resistance (AMR), vaccine development, vaccinology, mechanisms of antibiotic resistance

## Abstract

In the last century, life expectancy has increased considerably, thanks to the introduction of antibiotics, hygiene and vaccines that have contributed to the cure and prevention of many infectious diseases. The era of antimicrobial therapy started in the nineteenth century with the identification of chemical compounds with antimicrobial properties. However, immediately after the introduction of these novel drugs, microorganisms started to become resistant through different strategies. Although resistance mechanisms were already present before antibiotic introduction, their large-scale use and mis-use have increased the number of resistant microorganisms. Rapid spreading of mobile elements by horizontal gene transfer such as plasmids and integrative conjugative elements (ICE) carrying multiple resistance genes has dramatically increased the worldwide prevalence of relevant multi drug-resistant human pathogens such as *Staphylococcus aureus, Neisseria gonorrhoeae*, and *Enterobacteriaceae*. Today, antimicrobial resistance (AMR) remains one of the major global concerns to be addressed and only global efforts could help in finding a solution. In terms of magnitude the economic impact of AMR is estimated to be comparable to that of climate global change in 2030. Although antibiotics continue to be essential to treat such infections, non-antibiotic therapies will play an important role in limiting the increase of antibiotic resistant microorganisms. Among non-antibiotic strategies, vaccines and therapeutic monoclonal antibodies (mAbs) play a strategic role. In this review, we will summarize the evolution and the mechanisms of antibiotic resistance, and the impact of AMR on life expectancy and economics.

## Introduction

Throughout the evolution of our species, life expectancy has generally been between 25 and 35 years with infection diseases being the balance needle. The living conditions of human beings progressively improved to the point that nowadays, a person born in a high-income country can expect to live more than 85 years (1). This result has been achieved primarily by improved hygiene, antibiotics and vaccination introduction. These three pillars have represented the most effective medical interventions to reduce mortality and morbidity caused by infectious diseases. In fact, in the poor areas of our planet where hygiene and the use of antibiotics and vaccination are not well-established, life expectancy is still lower than 50 years (2). Vaccination has protected humans by training the immune system to recognize and establish a rapid and effective response against a pathogen. In the 20th century, vaccines were introduced to large populations with the immunization practice developed by Edward Jenner. Since then, vaccination has become a widespread medical treatment determining the control of 16 major diseases, namely, smallpox, diphtheria, tetanus, yellow fever, pertussis, *Haemophilus influenzae* type b disease, poliomyelitis, measles, mumps, rubella, typhoid, rabies, rotavirus, hepatitis B, pneumococcal, and meningococcal diseases. Noteworthy is the complete or partial eradication achieved for smallpox and poliomyelitis, respectively (3).

To counteract infectious diseases, the discovery of antimicrobial treatment was another significant milestone that has dramatically reduced mortality. The modern era of antimicrobial therapy initiated in the 19th century with the identification of anti-syphilitic and anti-trypanosomal molecules derived from organic compounds chemically synthetized (4). In 1928, the discovery by Alexander Fleming of a new class of non-toxic antimicrobial agents derived from environmental fungi gave rise to the “golden era” of antibiotic discovery (1945–1960) (5). Conversely to active vaccination, drugs are therapeutics with different modes of action targeting the bacterial functions such as cell wall integrity, nucleic acid synthesis and repair, or protein biosynthesis. Moreover, drugs can be naturally produced by microorganisms (including environmental fungi and saprophytic bacteria), generated by chemical modifications of the natural antimicrobial agents or fully synthetized (6).

**Figure 1:**
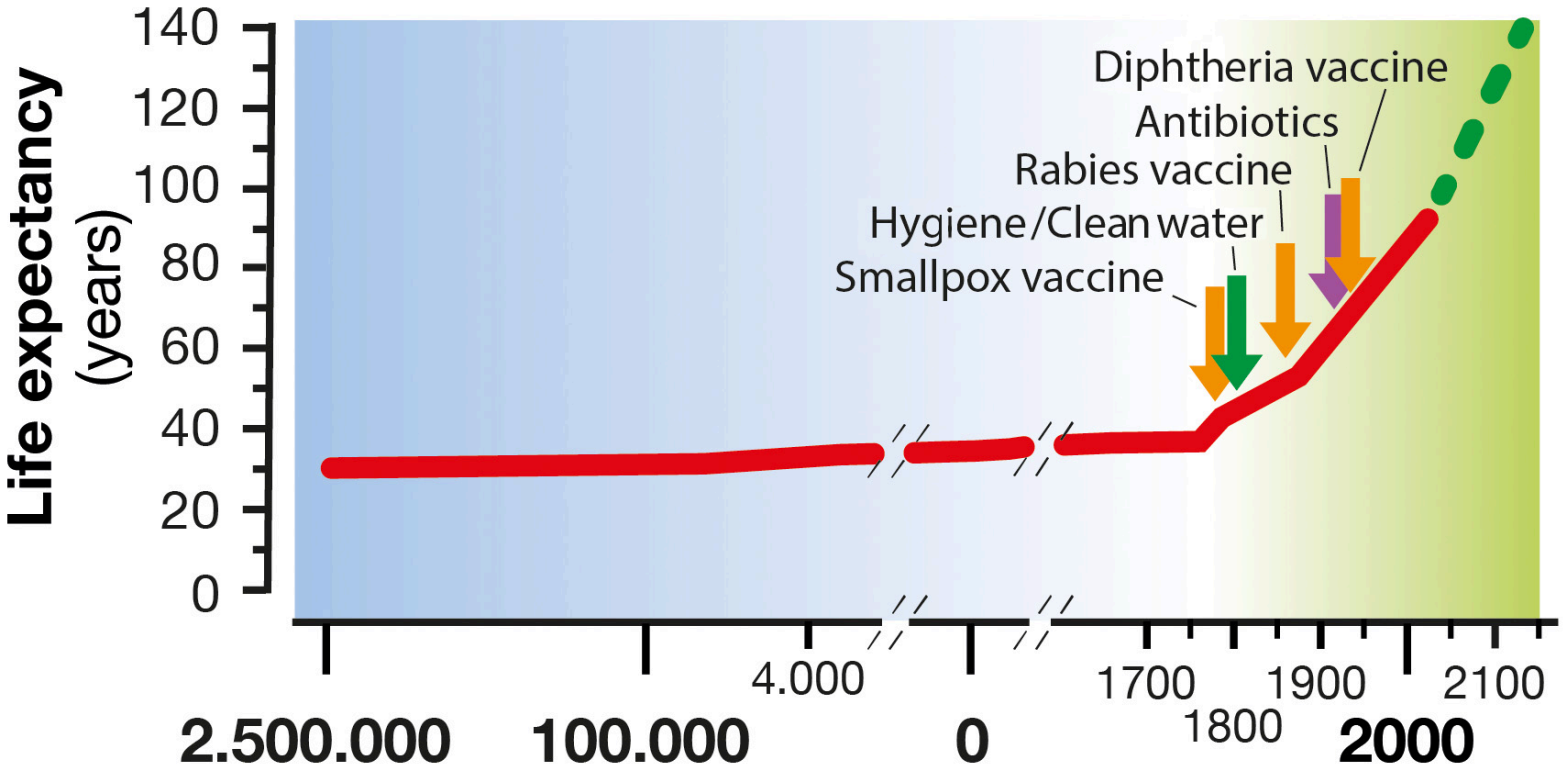
Life expectancy increase along human civilization. In the last century, life expectancy has increased considerably, thanks to the introduction of hygiene, clean water, antibiotics, and vaccines as a means of treatment and prevention of many infectious diseases.

**Figure 2:**
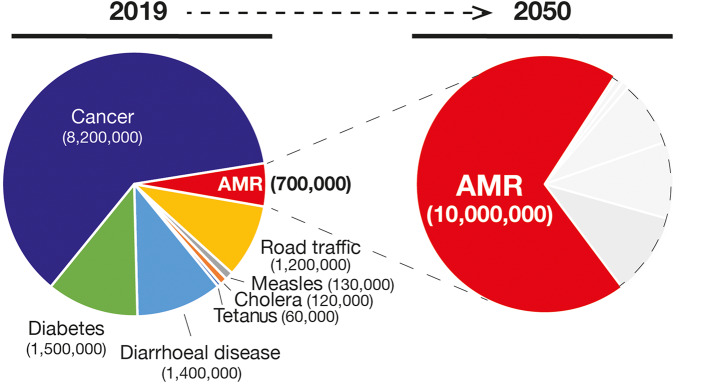
Number of deaths and the main causes **(Left)** in 2019 and the projection of number of deaths due to AMR infections in 2050 (in red in the **Right**). Gray areas represent other causes of deaths.

In combination with the vaccination practice, the discovery of antibiotics and their successful use in medicine is considered one the most relevant findings from a global health perspective (Figure 1). Nevertheless, the effectiveness of antibiotics has weakened to the point that our lives can be severely threatened. In fact, the antimicrobial resistance (AMR) is one of the most daunting problems that is causing the spread of infectious diseases and the increase in the number of deaths caused by infections that were previously considered uncomplicated (7). For example, the bloodstream infections caused by bacteria resistant to one or several drugs (multidrug-resistant; MDR) such as *Pseudomonas aeruginosa* are characterized by a 50% of mortality compared with the 24% of the non-multidrug-resistant infections (8). In addition, medical procedures such as surgeries, immunosuppressive chemotherapy and organ transplantation are becoming more critical and, in some cases, even prohibitive considering the need of effective antibiotics against multidrug-resistant pathogens. Therefore, the consequences of such microbial evolution can be dramatic with infectious diseases that could severely reduce our lifespan to an extent similar to the pre-antibiotic era. Globally, AMR pathogens are causing 700,000 deaths/year, and 10 million deaths/year are expected by 2050, a number even, higher than the 8.2 million caused by cancer today (9) (Figure 2).

## Antibiotic Resistance Mechanisms and Prioritization of Antibiotic Resistant Microorganisms

Antibiotic resistance is considered nowadays as one of the greatest threats to human health (10). Cases of antibiotic resistance are constantly reported, and the time needed for bacteria to become resistant to newly introduced antibiotics, is getting shorter. In fact, antimicrobial use exerts evolutionary pressure for the creation and transmission of resistant pathogens, thus reducing antimicrobial effectiveness and raising the incidence of severe disease (11). However, this is not a new phenomenon and is commonly observed as soon as the introduction of new classes of antibiotics occurs (12). In 1946, Alexander Fleming anticipated this global burden with the renowned sentence “There is probably no chemotherapeutic drug to which in suitable circumstances the bacteria cannot react by in some way acquiring “fastness” [resistance]” (13). In fact, penicillin became commercially available in 1943 and resistance was observed for *Staphylococcus aureus* by 1948. In this context, the discovery by Barbara McClintok that transposons play a major role in the genomic diversity and evolution paved the way for a deeper understanding of the genetic basis underlying the antimicrobial resistance dissemination. Transduction, conjugation, transformation and other mobile genetic materials (transposons and integrons) are all possible mechanisms for the transmission of genetic determinants involved in AMR (14, 15) and in the generation of “superbugs” (16). Worryingly, the transmission of multiple resistance genes is not only relatively common among organisms within the same genus but also among evolutionarily distant organisms.

**Figure 3:**
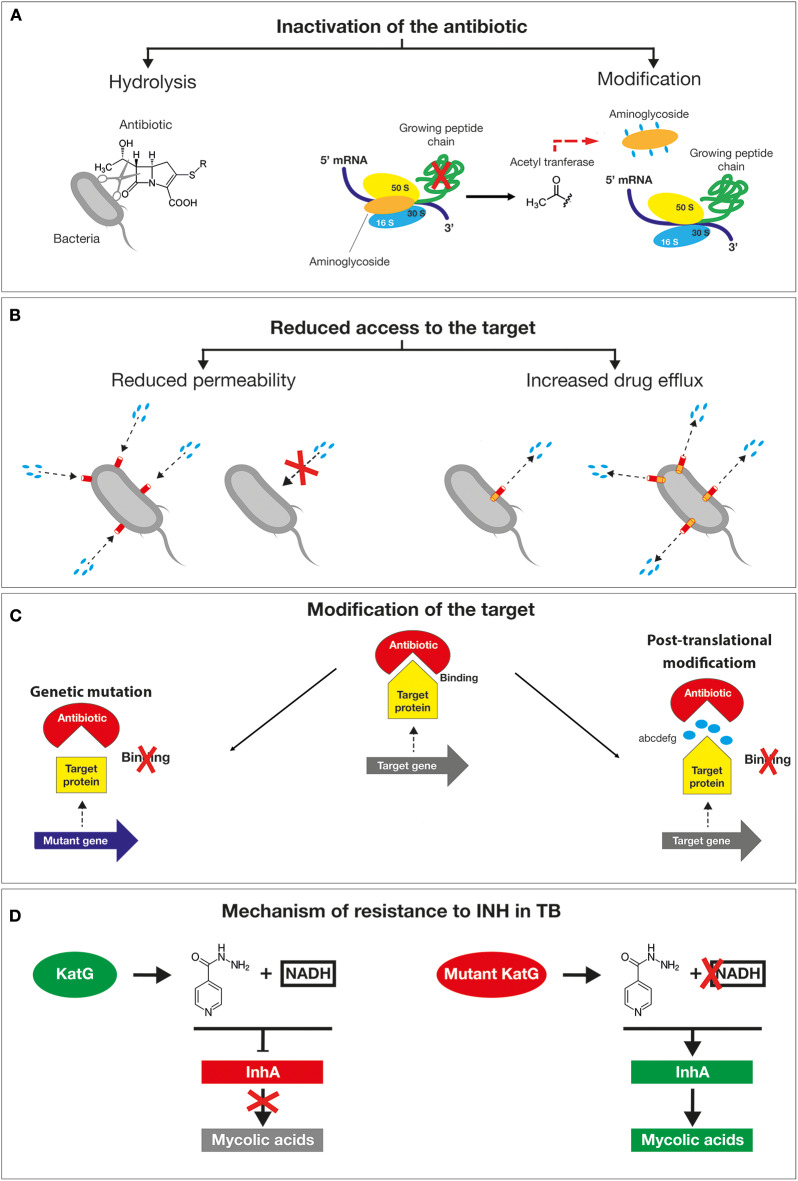
Schematic representation of the main mechanisms exploited by bacteria to tackle the action of antibiotics. **(A)** The inactivation of the drug by its hydrolysis or structural modification. **(B)** The prevention of the access to the target by reducing membrane permeability or overexpression of efflux pumps. **(C)** Changes in the antibiotic targets by mutation or post-translational modifications. **(D)** Resistance mechanism of *Mycobacterium tuberculosis* to isoniazid drug mutations in the *katG* gene causes the accumulation of the inactive form of the antibiotic inside the bacilli without the formation of active adduct.

A plethora of mechanisms has contributed to the emergence of antimicrobial resistant microorganisms and to their spread. The first classification is between natural and acquired resistance mechanisms. The natural resistance relies on the bacterial ability to block the antibiotic function as a result of inherent structural or functional characteristics. For example, vancomycin (glycopeptide antibiotic) which inhibits peptidoglycan crosslinking, is generally effective only against Gram-positive but not Gram-negative bacteria, due to its inability to cross the outer membrane to get access to the d-Ala-d-Ala peptides. By contrast, the acquired resistance is based on the gaining of new functions; bacteria that would be originally susceptible, became resistant to one or more antibiotics (17). The main mechanisms exploited by bacteria to tackle the action of antibiotics are based on (i) the inactivation of the drug by its hydrolysis or structural modification, (ii) the prevention of the access to the target by reducing membrane permeability or overexpression of efflux pumps, (iii) changes in the antibiotic targets by mutation or post-translational modifications (Figures 3A–C) (18). The mechanisms of antibiotics inactivation comprise both the production of enzymes that destroys the antibiotic itself (for example, β-lactamases) and the production of enzymes that modify its structure (for example, aminoglycoside-modifying enzymes). β-lactam antibiotics, such as penicillin and cephalosporins, are hydrolyzed by a wide variety of β-lactamases. Since the early discovery of these enzymes, bacteria resistant to all β-lactam antibiotics have emerged (19, 20). In this regard, the genes encoding for extended-spectrum β-lactamases (ESBLs with activity against oxyimino-cephalosporins) and carbapenemases (21), including the IMP (imipenemase), VIM (Verona integron encoded metallo β-lactamase), *K. pneumoniae* carbapenemase (KPC) and OXA (oxacillinase) have been identified not only on the chromosomes, but also on plasmids as in the case of *Enterobacteriaceae* (22, 23) and of *P. aeruginosa* (8). Among them, there is the New Delhi metallo-β- lactamase 1 (NDM-1), an enzyme that confers high level of resistance to multiple antibiotics, including penicillin, cephalosporins and carbapenems and that is capable of rapid worldwide dissemination (14). This is of high concern since there are few available treatment options beyond the carbapenems. The main producers of the NDM-1 enzyme are *Enterobacteriaceae* such as *Klebsiella pneumoniae, Escherichia coli* and *Proteus mirabilis*, but also *Acinetobacter* spp. and *Pseudomonas aeruginosa* (Figure 3A) (24). Lastly, aminoglycoside-modifying enzymes are able to modify the structure of the antibiotic molecule by the transfer of different chemical groups such as acyl, phosphate, nucleotidyl and ribitoyl groups. This modification prevents the antibiotic from binding to its target protein mainly due to the steric hindrance (Figure 3A). For example, in 2012 a novel genomic island encoding six aminoglycoside-modifying enzymes, including gentamycin, was identified in *Campylobacter coli* isolated from broiler chickens in China (25) and more recently in Group B streptococcus (26). Yet, the prevention of antibiotic access to a specific target can occur by reducing the permeability of the membrane and/or increasing efflux in order to reduce the intracellular concentration of a drug (27, 28). Differently from Gram-positive bacteria, Gram-negative bacteria are intrinsically less permeable to many antibiotics as their outer membrane forms a sort of permeability barrier. Generally, the permeability is reduced by the downregulation of the major porins, which results in a decrease in antibiotics entry into the bacterial cell. Porins are beta barrel proteins that cross the cellular membranes acting as channels for passive diffusion of certain molecules (29). The expression or the structure of a specific porin can be influenced by mutations that decrease membrane permeability, reducing the entry of antibiotics inside the cell. For instance, *P. aeruginosa* OprD-deficient strains are characterized by high level of resistance to carbapenems (30). Bacterial efflux pumps belonging to the resistance-nodulation-division (RND) family are not selective for a specific class of antibiotic and confer intrinsic resistance to multiple and structurally distinct classes of drugs, including the β-lactams, quinolones, and aminoglycosides (31). The high-level expression of efflux genes is often due to mutations in their regulatory network that generally occur within a local repressor, a global transcription factor, promoter regions or intergenic sites. For example, by mutations and gene duplication mechanisms the overexpression of MexAB-OprM multidrug efflux systems confers to some *P. aeruginosa* isolates the ability to became resistant to carbenicillin antibiotics. Moreover, Nicoloff et al. identified a large tandem duplication of the *E. coli* acrAB locus in a mutant isolated in the presence of tetracycline (32). Such a mutation was found to overexpress the AcrAB drug efflux pump, producing an MDR phenotype (33). In *N. gonorrhoeae*, a mutation in the consensus −10 sequence upstream of *mtrC s*trengthens its promoter activity determining constitutive overexpression of the efflux pump (34), (Figure 3B).

The other mechanism responsible for drug resistance is represented by the modification of the target by genetic mutations or post-translational modifications. Mutations in target genes can enable bacteria to overcome the toxic effects of antibiotics. Examples are provided by mutations in bacterial DNA gyrases (e.g. *gyrA*) and topoisomerase (e.g. *parC*) for fluoroquinolones, the β subunit of RNA polymerase (rpoB) for rifampin, 16S rRNA (rrs) for tetracyclines and aminoglycosides and 23S rRNA (rrl) for linezolid (13). The clinical use of linezolid has selected *S. pneumoniae* (35) and *S. aureus* (36) resistant lineages by a mutation in one of the rrl copies. The high recombination frequency has subsequently enabled the allele enrichment in the bacterial population. Another example of a target change is the acquisition of specific genes by horizontal gene transfer (HGT), such as in methicillin-resistant *S. aureus* (MRSA). In this case, the methicillin resistance is obtained by the acquisition of the staphylococcal cassette chromosome mec (SCCmec) element. This cassette encodes for a mutated form of PBP2 that is not recognized as a target by the β-lactamase enzyme but is still able to mediate a regular cell wall biosynthesis. Additional antibiotic resistance mechanisms consist in the protection by modification of the target molecule. This is exemplified by the polymyxin resistance in *Acinetobacter baumanni* (polymyxin B and polymyxin E, also known as colistin). The polymyxin antibiotics, are cyclic antimicrobial peptides with long, hydrophobic tails that bind to the lipopolysaccharide (LPS) of Gram-negative bacteria disrupting cell membranes. This resistance is often associated with changes in the expression of regulators affecting LPS production. For example, the addition of phosphoethanolamine (PE) to lipid A due to overexpression of *pmrC* results in a substantial reduction of colistin binding as the negative charge of the LPS is hugely lowered (37).

Given the alarming increase in antibiotic resistance cases, several human pathogens have been classified as a priority for the design of appropriate preventive and therapeutic strategies. In 2017, WHO published a prioritization list of pathogens (classified as critical, high and medium priority) to guide discovery, research and development of new antibiotic (38). In 2019 an estimation performed by the Centers for Disease Control and Prevention (CDC) in the United States reported that more than 2.8 million antibiotic-resistant infections occur in the U.S. each year, and more than 35,000 people die as a result of these infections. The antibiotic-resistant strains were classified into four categories according to the urgency of intervention (urgent, serious, concerning and watch list) (39). These bacteria can cause severe and often deadly infections and have become resistant to a large number of antibiotics, including carbapenems and third generation cephalosporins (the best available antibiotics for treating multidrug resistant bacteria). A relevant example is provided by *A. baumannii* multi-drug resistant isolates, that frequently cause outbreaks in hospitals. These strains are characterized by an extraordinary ability to rapidly alter their genome surviving on inanimate surfaces and medical equipments even in the presence of disinfectants (40). In *A. baumannii*, “resistance islands” located on the bacterial chromosomes and/or on mobile genetic elements induce a drug-resistant phenotype. For example, AbaR1 is an 86kb resistance- island generated by recurrent insertions of different mobile genetic elements, which can spread by horizontal gene transfer (HGT) within the genera *Pseudomonas, Salmonella*, and *Escherichia*. Genomic islands like AbaR1 confer resistance to various antibiotics by spreading the cluster of genes encoding for *OXA*-type beta-lactamases, aminoglycoside-modifying enzymes, proteins responsible for the modification of the lipid A and overexpression of the AdeABC efflux system (41). Tuberculosis (TB), even if not initially included in the WHO list, was already considered one of the most urgent priority because of its growing resistance to traditional treatment. TB now kills more people than any other pathogen (1.8 million in 2015) (42). Unfortunately, the resistance to isoniazid (INH), one of the four antibiotics used for the treatment of TB, is constantly increasing. INH leads to the inhibition of mycolic acids (MA) synthesis, the major lipid component of the mycobacterial cell envelope (43). The mechanisms of action of isoniazid are diverse and not fully known due to the involvement of a number of different genes. However, several studies suggest that the catalase KatG activates INH enabling the binding to NAD and the inhibition of enoyl acyl carrier protein (ACP) reductase (InhA). As a result, MA synthesis is blocked leading to mycobacteria death. When mutations in the *katG* gene occur, INH remains in an inactive form, unable to bind NAD and to inhibit InhA activity (Figure 3D). Consequently, InhA accomplishes its function in cell wall synthesis determining the survival of TB. This type of resistance is of concern as it negatively affects the outcome of TB-treatment facilitating the dissemination of MDR-TB and reducing the efficacy of INH (44). The second and third tiers in the list—the high and medium priority categories—include other increasingly drug resistant bacteria that cause more common diseases, such as *Neisseria gonorrhoeae* (the causative agent of gonorrhea) (45, 46). Since the treatment of *N. gonorrhoeae* with sulfonamides, this bacterium has acquired genetic resistance determinants capable to inhibit the killing by all major classes of antibiotics that are used as first line methods of treatment against gonorrhea infection (47). The main lethal target of cephalosporins is the transpeptidase penicillin binding protein 2 (PBP2), encoded by *penA*. Most of the resistant isolates contain mosaic mutations in *penA* that confer resistance to cephalosporins. In addition, the overexpression of efflux pump, MtrCDE, contributes to resistance through an increase of the drug efflux. The simultaneous mutations in the major porins PorB and mtrR, the repressors of MtrCDE efflux pump, represent an important resistance determinant of gonococcus. Hence, the outstanding capacity of *N. gonorrhoeae* to rapidly acquire resistance has led the World Health Organization and Center for Disease Control (US) to classify this bacterium as “superbug.” As a result, if new prophylactic and/or therapeutic strategies will not be discovered soon, we may enter an era of incurable gonorrhea infections (48). Therefore, understanding the mechanisms underlying the AMR will help to rationally design new preventive strategies to effectively tackle these dangerous threats for the human health.

## Evolution of Drug Resistance: What Did We Do Wrong?

Antibiotic resistance occurs when bacteria are not controlled nor killed by previously effective drugs through several mechanisms described above. Resistance is largely driven by the abuse of antibiotics in humans and animals, often with little or no therapeutic benefit. The use of antibiotics in viral respiratory infections or abuse in animal care and in agriculture to promote livestock growth, are common examples of the misuse (49). Additionally, the inability to rapidly identify the specific infecting pathogen at the point of care make the empiric treatment the only possible choice. In fact, the empiric treatment requires the use of broader spectrum antibiotics to cover all the key pathogens responsible for a particular infection (50). Moreover, the relationships between the human microbiome and the host immune system is hugely threatened. Antimicrobials alter the structure of the microbiota, expand the pool of antimicrobial-resistance genes, degrade the protective effects of the microbiota against invasion by pathogens, and may impair the efficacy of alternative medical treatments (51). Hence, the abuse of antibiotics has negatively affected the immune response making individuals more susceptible to infections. In fact, the pressure imposed by the antibiotic treatments on microbes other than the targeted pathogen (“bystander selection”) is hypothesized to be a major factor in the propagation of antibiotic resistance (11). International traveling is also a key factor in the dissemination of highly resistant strains. New Delhi metallo-β- lactamase 1 (NDM-1) and *mcr-1* are the mobile resistance genes increasingly spreading worldwide and generating resistance to a variety of antibiotics including penicillins, cephalosporins and carbapenems. This is particularly relevant for infections caused by Gram-negative bacteria sensitive only to carbapenems, and for which only very few alternative possibilities are available. Furthermore, the NDM-1 resistance genes are transmitted very easily, as shown by the two Dutch travelers which were infected by a NDM-1-producing bacterial strain during their trip to India, even if not visiting any health centers (52). *Enterobacteriaceae* (*Klebsiella pneumoniae, E. coli* and *Proteus mirabilis*), *Acinetobacter* spp. and *Pseudomonas aeruginosa* are the most common NDM-1-producing bacteria for which colistin is considered the only possible therapeutic option. Nevertheless, with the spread of the *mcr* resistance gene (*mcr-1*) a generation of pan-resistant strains is arising. This has been probably due to the large use of colistin to increase pig growth in farms in China. In addition, to the raw meat, *mcr-1* has been isolated also in samples from infected patients (53). The *mcr-1* resistance determinant is able to easily spread in different bacterial species and also worldwide due to business globalization (24). Hence, the spread of resistant organisms, highly infectious and inducing high rate of morbidity and mortality has a strong economic impact, in terms of costs of treatment and long-term hospital stay, with a great burden on health systems.

## Vaccine Against Antibiotic Resistance: A Valuable Weapon To Fight AMR

The rising of AMR is a threat to modern medicine and new measures to control antibiotic-resistant bacteria are desperately needed including the development of new antibiotics. Nevertheless, the discovery of new effective chemical compounds with an appropriate balance of antibacterial activity, drug metabolism, pharmacokinetics properties and safety it is a daunting task. In fact, the pipeline run dry ~40 years ago (54). In addition, challenging logistics and high costs of large clinical trials make them nearly impossible to bring to market. Even if successful, the clinical utility of antibiotics will decline as resistance to them inevitably rises (55). Improving hygiene and the correct use of antibiotics while expanding their access in low- and middle-income countries are important tools to limit the burden of AMR, despite the fact these measures alone are not enough (50). Hence, vaccines may become a valuable and effective weapon to fight AMR. An important aspect is that resistance mechanisms are of less concern in vaccination. As reported above the mechanisms of resistance to antibiotics are mediated by the generation of spontaneous mutations or the acquisition of mobile genetic element by HGT. These mechanisms confer to bacteria the ability to tackle the killing effect of a drug and survive. Antibiotics are therapeutic measures since generally prescribed after the settling down of an infection when hundreds of millions of bacteria are infecting the body. On the contrary, vaccines are designed to prevent diseases. Their prophylactic use allows the host to build an immune response before encountering the pathogen or even at the beginning of an infection when only a few hundred or thousands bacteria are present. As the occurrence of resistance mechanisms can stochastically arise among billions of bacteria, it is evident that this is less likely to occur following vaccination. Furthermore, most antibiotics have a single target while vaccines have multiple targets inducing host-specific antibody and/or T cell responses. Even in this case, more mutations are likely needed to confer resistance to vaccines making the development of microbial resistance even harder (56, 57). Therefore, vaccines can be effective against antimicrobial resistance in different ways:

(I) By lowering the inappropriate use of antimicrobial compounds. Perhaps counterintuitive is the evidence that viral vaccines are also very effective in reducing AMR. For istance, vaccines against influenza virus reduce the incidence of fever and sickness which affect a significant proportion of community-dwelling elderly population each year in the US (58). By preventing a proportion of these cases, vaccines can reduce both, the inappropriate use of antibiotics prescribed in case of viral infections and the need of antibiotic treatment to cure secondary bacterial infections (59).
(II) By reducing the insurgence of resistant serotypes. For example, the pneumococcal polysaccharide conjugate vaccines had an effect of direct protection of infants and of herd immunity in adults initially not targeted by routine immunization (60). Remarkably, also the antibiotic prescriptions and the prevalence of antibiotic resistant strains decreased. In the 1990s, before the introduction of a 7-valent pneumococcal conjugate vaccine (PCV7), more than 63,000 cases of invasive pneumococcal disease occurred each year in the US. Between 2000 and 2004, a 57% reduction in the incidence of penicillin-non-susceptible invasive pneumococcal disease (IPD) and 84% reduction in the rate of multidrug-resistant strains were achieved. These data indicate that vaccination is effective, regardless of the bacterial resistance phenotype. However, the universal use of PCV-7 led to increased prevalence of serotype 19A, a non-vaccine serotype with high rate of penicillin resistance (61). Introduction of 13-valent PCV in 2010, which contains 6 additional serotypes, including 19A, further reduced the incidence of IPD and of antibiotic-resistant pneumococci. Nevertheless, the risk of the evolution of AMR in pneumococcal serotypes not contained in the vaccine is still high. In this context, the design of a vaccine aimed to specifically target resistance determinants or resistant strains is highly valuable (62).
(III) By reducing infection rate of resistant strains in closely related species. Between 2004 and 2008, in New Zealand, 1 million people were vaccinated with the Outer Membrane Vesicles (OMV) based vaccine (MeNZB) to fight a meningococcus B outbreak. A retrospective case-control study has shown that the immunization with MeNZB resulted in 31% reduction of *Neisseria gonorrhoeae* infection (63). *N. gonorrhoeae* is one of the most common bacterial sexually transmitted diseases (STDs), with ~100 million cases worldwide. One of the main concerns of gonococcal infection is the emergence of strains resistant to nearly all classes of antibiotics including the expanded-spectrum cephalosporins and the lack of an effective vaccine. Hence, *N. gonorrhoeae* infections are becoming as the most prevalent and difficult to treat. Despite causing very different diseases, *N. meningitidis* and *N. gonorrhoeae* share 80–90% nucleotide identity at the genome level. Therefore, it is reasonable to assume that antibodies against common antigens could induce a cross-protective effect (64). Bioinformatic analyses has revealed that 57 OMPs are of *N. meningitidis* are conserved also in 970 *N. gonorrhoeae* strains isolated in US (65). Among them, PilQ, Omp85 (BamA), NspA, MtrE, MetQ, and LbpA show 93% amino acid sequence similarity to *N. gonorrhoeae*, suggesting their potential contribution to cross-protection (64). Further investigations are needed to evaluate the effectiveness of MenB-4C vaccine in preventing gonococcal infections (66).
(IV) By directly targeting antibiotic resistant microorganisms. Although many different vaccine formulations have been proposed to prevent infection by antimicrobial resistant pathogens, (such as *S. aureus, E. coli, Clostridium difficile* etc.) no successful phase III clinical trial data have been published yet (67, 68). A possible reason for the failure in developing vaccines against these pathogens are the multiple virulence mechanisms a vaccine should target, as well as the absence of animal models that are representative of human diseases (69, 70). A better understanding of host-pathogen interactions such as immune evasion, and increased knowledge in the epidemiology and variability of the main antigens, could help in the development of novel effective vaccine. However, new incentives may be necessary for the development of novel vaccines leading to the refinement of health care system against AMR infections and eventually saving millions of lives.

## Modern Technologies and The Future of Vaccines Against AMR

### Live-Attenuated or Killed Microorganisms and Subunit Vaccines

Attempts to “vaccinate” begin even before the Edward Jenner's smallpox vaccination was introduced. In the 7th century, some Indian Buddhists drank snake venom in an attempt to become immune to its effect. The process of variolation, consisting in the introduction of dried pus from smallpox pustules into the skin of a patient, was practiced at regular intervals by the Brahmin caste of Hindus in India in the 16th century (71). Nevertheless, Edward Jenner is considered the real father of vaccinology; in 1796 he tested the protective effect of a prior cowpox infection against smallpox by actively immunizing an eight-year-old-boy with cowpox and then carrying out smallpox challenge (variolation) (72). In the 19th century, Louis Pasteur gave a fundamental contribution in the field with the creation in laboratory of the first vaccine. The main principle of Pasteur was to “isolate, inactivate and inject” the causative agent of a disease. His rationale was to attenuate or inactivate the virulence of the disease-causing organisms while preserving their immunogenicity (5). Live attenuated vaccines (LAV) have been available since the last century and derive from viruses or bacteria that have been weakened under laboratory conditions, so that they induce immunity by simulating natural infections causing no or very mild disease (73). Moreover, they provide a recurrent antigenic stimulation that ensures memory cells production. An example is provided by the TB treatment with the Bacillus Calmette-Guérin (BCG) developed in 1922. Killed microorganisms are safer, do not induce disease and have an excellent stability profile, even if the immune response that they induce is reduced in terms of magnitude compared to LAV and several doses of vaccine may be required. For many viral diseases, the risk of reinfection is null due to the induction of antibodies directed against invariable antigens (e.g., smallpox, yellow fever, polio, rabies, mumps, measles, rubella, varicella, herpes zoster, hepatitis A, and Japanese encephalitis). Therefore, vaccines that were developed following the principles described in this paragraph have proven to be successful (74). A further development of Pasteur's strategy was the so-called “subunit” vaccine that contains only the antigenic determinants that are able to induce an effective immune response. This approach has been successfully applied to diphtheria and tetanus, two diseases caused by bacterial toxins (75).

### Recombinant DNA

The development of safer live-attenuated vaccines and subunit vaccines through rational design has been possible when in the 1980s recombinant DNA techniques were introduced and revolutionized the field of vaccinology. This new approach enabled the generation of the genetically detoxified Bordetella pertussis toxin (76) and the recombinant hepatitis B surface antigen (HBsAg) (77). Genetic engineering has also been applied to generate attenuated viral and bacterial strains as antigen and delivery system for heterologous antigens, or to develop genetic “reassortants,” as in the case of the influenza attenuated vaccine or the rotavirus vaccine, which is based on a bovine rotavirus “reassorted” with 5 human rotavirus strains (78).

### Glycoconjugate and Bioconjugate

In the previous decades, innovative polysaccharides-based vaccines have been developed to protect against infections caused by *Neisseria meningitidis, Streptococcus pneumoniae*, and *Haemophilus influenzae* type b (Hib). However, these vaccines were not immunogenic in children below 2 years of age and they induced a short memory response. To enhance immunogenicity, an effective strategy was to covalently link the polysaccharide antigen to a carrier protein, thereby providing helper T-cell activation in addition to the B-cell dependent immune response. Examples of protein carriers that have been efficiently used for the chemical conjugation are tetanus toxoid (TT), diphtheria toxoid (DT), and a non-toxic cross-reacting mutant of DT (CRM197). The resulting glycoconjugate vaccines elicited a more potent functional immune response in all age groups. Nowadays, this type of vaccines are available for the meningococcus serogroups A, C, W, Y, and *H. influenzae* type b (Hib) and Pneumococcal vaccines. The glycoconjugate vaccines have been able to successfully prevent the deaths and hospitalization of millions of people during the past three decades (79, 80). However, the requirement of complex manufacturing makes the process of glycoconjugation expensive and unsuitable for very complex vaccines, or for their use in low-income countries. These limitations can be overcome by a process called “bioconjugation,” a new platform technology to generate glycoconjugate vaccines *in vivo* in a single step, drastically simplifying glycoconjugates development and manufacture. Bioconjugation is based on recombinant non-pathogenic *Escherichia coli* strain that co-express (i) a heterologous, antigenic polysaccharide, (ii) a carrier protein encoding the glycosylation sites, and (iii) an enzyme covalently coupling both substrates during bacterial growth. Clinical trials with either mono- and multi-valent vaccine candidates produced using the PglB conjugating enzyme showed safety, immunogenicity and functionality of the immune response (81, 82). Bioconjugates may play a fundamental role in the future to prevent infection caused by dangerous AMR pathogens expressing polysaccharide antigens, such as those reported in the WHO and CDC priority lists (38, 39).

### Adjuvants

A limitation of many vaccines is the poorly immunogenic response they evoke. Thanks to the advances in the knowledge of mechanisms underlying innate immunity, effective vaccine adjuvants have been developed to enhance the immune systems' response. (83). These compounds are able to improve the speed, potency, and persistency of the immune response to vaccination. While aluminum was the first and only adjuvant routinely used in human vaccines for more than 50 years, novel adjuvanted system (AS) composed of the combination of two or more different immunostimulants were developed to achieve the desired response. For example, one of them, namely AS04, was used in 2005 for the hepatitis B (HBV) vaccine and in 2007 for the human papillomavirus HPV-16/18 vaccine (84). More recently, AS01, an adjuvant composed by two immunostimulants, (saponin QS21 and monophosphoryl lipid A, targeting TLR4), has been licensed for a vaccine against malaria and herpes zoster vaccine. Also, it is currently used in clinical trials for vaccines designed to prevent reactivation of tuberculosis, and for a vaccine against non-typeable *H. influenzae* and *Moraxella catarrhalis*, aimed to prevent exacerbation in chronic obstructive pulmonary disease. The TB vaccine phase 2b trial conducted in Kenya, South Africa, and Zambia provided 54.0% protection in *M. tuberculosis* infected adults against active pulmonary tuberculosis disease. This vaccine is composed of the M72 recombinant fusion protein including two immunogenic *M. tuberculosis* antigens (Mtb32A and Mtb39A), combined with the AS01_E_ adjuvant system (85). Additional adjuvants targeting the innate immune receptors TLR9 (DNA oligonucleotides), TLR5 (Flagellin), TLR7, surface-exposed (TLR1/2), endosomal (TLR3), or cytoplasmic (RIG I, DNA sensors) innate immune receptors have been successfully tested (83).

### Reverse Vaccinology, Generalized Modules for Membrane Antigens (GMMAs) and Nanoparticles

A new era in vaccine design is started through the use of the bacterial genomes to discover new vaccine candidates. “*in silico*” prediction tools are applied to identify genes encoding for surface-exposed or secreted antigens. Newly discovered antigens are expressed in *E. coli* as recombinant proteins, and their immunogenicity evaluated in preclinical models (86). This approach, named “reverse vaccinology” has allowed the development of the first multivalent protein-based vaccine against meningococcus B, 4CMenB, that is now approved in many countries worldwide. In addition to the recombinant proteins identified by the reverse vaccinology approach, the licensed 4CMenB vaccine contains the highly immunogenic outer membrane vesicles (OMV) component produced through detergent extraction (87). The same OMV component has been used in New Zealand to fight a MenB outbreak. An immunization campaign was implemented in all population starting from 5 weeks to 20 years of age in the period 2004–2006 (64, 88, 89). Interestingly, by using genetic approaches, it is possible to induce an over-blebbing phenotype without using detergents leading to a new generation of improved bacterial OMVs called generalized modules for membrane antigens (GMMAs). The vesicles are obtained by mutation in genes responsible for stabilizing the link between the bacterial outer membrane and peptidoglycan (e.g., *tolR, tolB, nlpl*). GMMAs can also be further engineered by targeting genes responsible for LPS acetylation, such as *lpxlM* and *lpxL1* therefore reducing LPS reactogenicity (90). Yet, another class of vaccines proposed to overcome the limitations of conventional and subunit vaccines is based on nanoparticles. In this regard, thanks to advancement in structural biology the rational design of molecule structures with desired size, shape, stability, improved immunogenicity and functionality became feasible (91). Nanoparticles were initially discovered in virus such as those from Hepatitis B. Once produced recombinantly these particles were found to be composed either of the surface antigen (HBsAg) or the core antigen (HBcAg). The HBcAg was shown to be assembled into particles of 24–31 nm diameter resembling the highly immunogenic viral cores obtained from HBV-infected human liver. Other class of self-assembling protein nanoparticles has been identified from a wide variety of source. For example, one of these is represented by a 24 subunits molecule, each composed of a four-alpha-helix bundle, that self-assembles in a quaternary structure with octahedral symmetry named Ferritin. DNA technology can be also used to construct genes that encode for self-assembling polypeptides fused with the desired immunogenic epitope for expression of the chimeric molecule in a selected cell expression system. The chimeric polypeptide then self-assembles within the cell, with an ordered pattern of surface exposed epitopes (92). An important advantage of this approach is that proteins of interest are exposed on the surface of the nanoparticle in the correct conformation thus eliciting a high specific immune response.

### Reverse Vaccinology 2.0 and Structural Vaccinology

Reverse Vaccinology 2.0 is aimed at the identification of antigens inducing high functional antibodies (93). By single B cells sorting and culturing, antibodies (Ab) with the desired functionality are selected and the corresponding Ig gene sequenced, and Abs produced as recombinant proteins. Three-dimensional structure resolution of the Ag–Ab (Fab) complex leads to a detailed definition of the protective epitope. Structural information and identification of the protective epitopes can drive the design of a novel optimized immunogens (“structure-based Ag design”). The new Ag can then be included in the best formulation or delivery system and tested in humans.

The Reverse Vaccinology 2.0 approach has allowed the identification of the cytomegalovirus CMV pentameric complex (94) and of the pre-fusion of F protein of the respiratory syncytial virus (RSV) (95). Although this approach has been exploited for viral pathogens, it is expected that the same technologies may also be applied to bacterial pathogens. Growing knowledge in this field could lead to rational design of new antigens more stable and able to elicit high level of functional antibodies. An example of structure-based design of bacterial antigens is the factor H binding protein (fHbp) of *Neisseria meningitidis*. This is a key meningococcal vaccine which exists as more than 300 peptides that can be grouped into three variants that do not elicit cross-protective immunity. Definition of the 3D structure and the mapping of all protective epitopes has guided the design of a new chimeric molecule containing combination of the main epitopes of the three variants but maintaining the overall conformation and the same structural features. The new chimeric fHbp was able to induce, in mice, cross-reactive immunity against the three variants (96).

### Monoclonal Antibodies

An alternative strategy proposed to combat AMR infection is based on the use of monoclonal antibodies. The main modes of action of anti-bacterial antibodies are based on the neutralization of virulence factor activities and inhibition of complement-mediated bacterial lysis. An important aspect is that the efficacy of monoclonal antibodies is dependent upon the expression level and the degree of sequence conservation of the corresponding target epitope. Hence, the ideal epitope should be highly conserved in structure and to be easily accessible for antibody binding. This is the case of monoclonal antibodies against bacterial toxins approved for clinical use which include two antibodies against anthrax (97, 98) and one targeting the toxin B of *Clostridium difficile*, approved for the prevention of recurrent infections in high-risk patients (99). As described above, the new technologies could allow the analysis of immune response and the identification of human monoclonal antibodies naturally produced by infected or vaccinated human donors. This will guide the design of novel and highly immunogenic antigens and antigen derived peptides. Analyzing the Ab-Ag complex, it would be possible to design powerful antigen molecules with high neutralization activity as in the case of the RSV preF antigen (95). The outstanding power of these new technologies is boosting our ability to identify, design and stabilize antigens in a very short time frame and consequently starts clinical trials immediately after (100). Effective medical treatments are urgently needed to address major global threats, and in this context, monoclonal antibodies represent a unique valuable weapon. In developing human monoclonal antibodies careful attention should be paid to evaluate possible reactions with human tissue at early preclinical stages. Reactions can be limited by using fully human monoclonal antibodies and by an accurate selection of the targeted epitopes.

### RNA Vaccines

RNA-based vaccines can be divided into non-replicating and self-amplifying mRNA vaccines (SAM) both of which make use of the host cell translational machinery to produce the selected antigens. More in detail, SAM-based vaccines consist of an engineered RNA viral genome encoding nonstructural proteins (nsPs) in which the genes encoding for the structural protein are replaced with the antigen of choice. RNA vaccines have shown to be effective in case of viral pathogens; however, this technology could be successfully applied also to bacterial AMR targets. Interestingly, a SAM vaccine encoding for a double-mutant of Streptolysin-O (SLOdm) and the backbone protein of pilus island 2a (BP-2a) from Group A (GAS, Streptococcus pyogenes) and Group B (GBS, Streptococcus agalactiae) was shown to be immunogenic in mice inducing both humoral and cellular immunity (101). Differently from viral vectors and DNA -based vaccine, RNA vaccines do not interact with the host-cell DNA, avoiding the potential risk of genomic integration and should not induce anti-vector immunity. Therefore, based on (i) their fully synthetic nature, (ii) relatively low production cost, and (iii) the possibility of expressing complex antigens in large amounts and in a short time, mRNA technologies could represent a rapid and valuable platform to be further exploited for vaccines against AMR infections.

**Figure 4:**
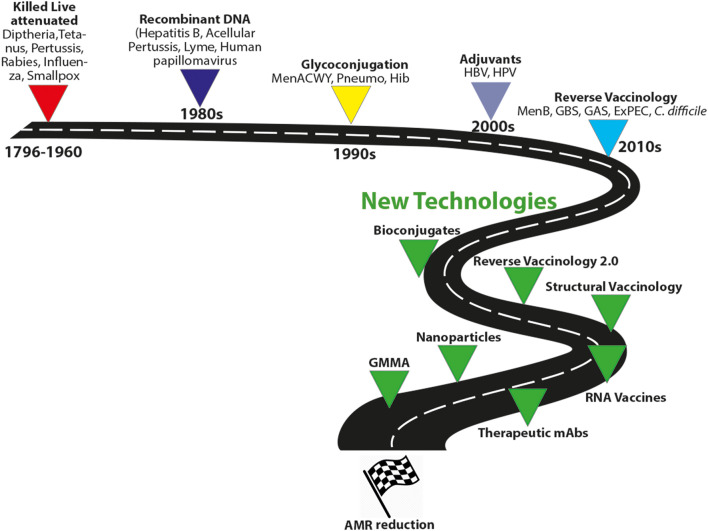
Evolution of vaccine technologies and platforms. MenACWY, meningococcus ACWY; Pneumo, pneumococcus; HBV, hepatitis B virus; Hib, type B *Haemophilus influenzae*; GBS, group B Streptococcus, GAS HPV, human papillomavirus; MenB, meningococcus B; GAS, group A streptococcus; ExPEC, extraintestinal pathogenic *E. coli*; Generalized Modules for Membrane Antigens, GMMA.

Altogether, these new technologies may greatly advance the field for the control of AMR infections (Figure 4).

## Conclusions and Future Perspectives

Rising antimicrobial resistance (AMR) is a threat to modern medicine and it also represents a major economic burden on healthcare systems. In the European Union, antibiotic resistant infections determine an economic burden of ~€1.5 billion per year. The US health system spends 20 billion USD in excess each year to treat resistant infections (102). By 2050, it is foreseen that USD100 trillion will be the economic damage due to AMR. According to the World Bank simulation, the global economy could lose as much as 3.8% of its annual gross domestic product by 2050 in a worst-case scenario. However, estimating the incidence and attributable mortality is challenging. This cost depends on a wide variety of factors: which drug and pathogen are involved, the mechanism of antibiotic resistance, the prevalence of that pathogen, the types of infections, the level of transmissibility, the health burden of those infections and whether alternative treatments are available. AMR can result in treatment failures and previously uncomplicated infections become more complex and severe (103, 104). It has recently been estimated that a 30% reduction in the efficacy of antibiotic prophylaxis for surgical procedures and chemotherapy would result in 120,000 additional infections and 6,300 infection-related deaths per year only in the United States. There is a risk of the cost being far higher than current best estimates. As already mentioned, vaccines represent a valuable and extraordinary tool to tackle the AMR problem. Besides being able to protect against serious diseases, vaccines may also reduce avoid mild disease episodes that may not receive medical attention, but which have important societal consequences. Vaccination decreases the disease incidence, the number of deaths, the longer hospital stays, and the need for more antibiotics if others fail owing to resistance. Unvaccinated individuals also benefit from wider societal benefits such as the lower transmission rates. This can be translated into a reduced risk of epidemics. In health economics, Incremental Cost–Utility Analysis (ICUA) is a method of financial analysis that can drive procurement decisions. The purpose of ICUA is to perform a cost-effectiveness analysis based on a careful evaluation of the ratio between the cost of an intervention and the benefit it generates in terms of increase in life expectancy. Value of vaccines and drugs is calculated based on the ICUA financial method resulting in an underestimation of the former. The added value of vaccines such as the induction of herd protection, reduction in time of hospitalization and reduction in anti-microbial resistance are difficult to measure in the short term. Therefore, since in the ICUA analysis, the benefits of medical interventions are evaluated during the period of medical observation, important benefits of vaccines which are only measurable in the long term are not considered. Although the ICUA analysis has generated more comprehensive models for financial evaluation, all the benefits of vaccination still need to be included in the analysis for the measurement of a full vaccine value (105). In 2015, the burden of resistant infections in countries of the European Economic Area (EEA) was measured in terms of number of cases, attributable deaths, and disability-adjusted life-years (DALYs) providing useful information for public health decision-makers prioritizing interventions for infectious diseases (106). The contribution of various antibiotic-resistant bacteria to the overall burden varies greatly between countries, thus highlighting the need for prevention and control strategies that are tailored to the needs of each country. Evaluation of the economic cost of AMR is important for decision-making and should be estimated accurately. Only combined efforts in the fields of antibiotics, new technologies for vaccine development and monoclonal antibodies will provide affordable solutions to tackle antimicrobial resistance.

## Author Contributions

SN, RRo, MP, and RRa wrote the manuscript and contributed to the ideas and concepts it contains. All authors reviewed and approved the manuscript.

## Conflict of Interest

RRo, MP, and RRa are permanent employees of the GSK group of companies. RRo, MP, and RRa are listed as inventors on patents owned by the GSK group of companies. SN is a PhD Research Fellow of University of Bologna (IT). This work was sponsored by GlaxoSmithKline Biologicals SA.
