# Corrigendum: Vaccines Against Antimicrobial Resistance

**DOI:** 10.3389/fimmu.2020.01578

**Published:** 2020-07-21

**Authors:** Roberto Rosini, Sonia Nicchi, Mariagrazia Pizza, Rino Rappuoli

**Affiliations:** ^1^GSK, Siena, Italy; ^2^Department of Pharmacy and Biotechnology (FaBiT), University of Bologna, Bologna, Italy; ^3^vAMRes Lab, Toscana Life Sciences, Siena, Italy; ^4^Faculty of Medicine, Imperial College London, London, United Kingdom

**Keywords:** vaccines, infectious diseases, antimicrobial resistance (AMR), vaccine development, vaccinology, mechanisms of antibiotic resistance

In the published article, there was an error regarding the affiliation for Mariagrazia Pizza. The author was incorrectly assigned affiliation 2 “Department of Pharmacy and Biotechnology (FaBiT) University of Bologna, Bologna, Italy,” though it should be affiliation 1 “GSK, Siena, Italy.”

In addition, the name of the institution in affiliation 4 was incomplete. The affiliation was captured as “Faculty of Medicine, Imperial College, London, United Kingdom,” though it should be “Faculty of Medicine, Imperial College London, London, United Kingdom.”

The authors apologize for these errors and state that this does not change the scientific conclusions of the article in any way. The original article has been updated.

